# Supplementary material for: Effect of external therapies of traditional Chinese medicine on constipation in patients with CRF: A meta-analysis
Source: PLoS One. 2023 Oct 5;18(10):e0291968. doi: 10.1371/journal.pone.0291968 (PMC10553223; doi:10.1371/journal.pone.0291968)
Supplement: Attachment — Submitted filename: PONE-D-23-04019_reviewer 20.4.23.pdf [file pone.0291968.s004.pdf]

PLOS ONE

Effect of External Therapies of Traditional Chinese Medicine on Constipation in Patients with CRF: A Meta-Analysis

--Manuscript Draft--

**Manuscript Number:**	PONE-D-23-04019
**Article Type:**	Research Article
**Full Title:**	Effect of External Therapies of Traditional Chinese Medicine on Constipation in Patients with CRF: A Meta-Analysis
**Short Title:**	Effect of External Therapies of Traditional Chinese Medicine on Constipation in Patients with CRF
**Corresponding Author:**	Wu Yu<br>Hangzhou First People's Hospital<br>Hangzhou, CHINA
**Keywords:**	
**Abstract:**	Objective The purpose of this study was to evaluate the curative effect of external therapies of traditional Chinese medicine in chronic constipation and to provide scientific theoretical basis for clinical practice. Method We searched the English database of PubMed, EMBASE, the Cochrane Library and the Web of Science and Chinese database of CNKI, Wan fang database, VIP Database and China Biomedical Literature Database up to December 2022. Randomized controlled trials (RCTs) involving constipation in patients with CRF that compared external therapies of traditional Chinese medicine and routine treatment to routine treatment were eligible for the analysis. A meta-analysis of the outcome indicators including total efficiency, weekly defecation times, defecation time, defecation difficulty score, patient-assessment of constipation quality of life and adverse events of treatment were performed. The analysis was performed by using Review Manager version 5.3. Result A total of 23 studies were included，with 1764 patients. Meta-analysis results showed that compared with the control group，the test group could significantly increase weekly defecation times(MD = 0.94，95%CI（0.70，1.18） ，Z=7.74，P < 0.00001), reduce defecation time(MD = -2.92，95%CI（-3.69，-2.16） ，Z=7.49，P < 0.00001), reduce defecation difficulty score(MD = -1.92，95%CI（-2.45，-1.39） ，Z=7.11，P < 0.00001), improve the quality of life in patients with constipation(MD = -7.57，95%CI(-10.23，-4.91)，Z=5.58，P < 0.00001) and obtain a higher total effective rate of treatment(OR=4.53，95%CI（3.27，6.29） ，Z=9.07，P < 0.00001). In terms of safety, there was no significant difference in the incidence of adverse events between two groups(OR=0.36，95%CI（0.04，2.95） ，Z=0.95，P =0.34). Conclusion The combination of external therapies of traditional Chinese medicine and routine treatment could achieve an excellent curative effect, and there was no specific adverse event. However because of the limited level of current evidence, more high-quality trials are needed in the future.
**Order of Authors:**	Wu Yu
	Qisu Ying
	Yajing He
	Xiangcheng Xie
	Xiao Yuan
	Ming Wang
	Xiao Fei
	Xiu Yang
**Additional Information:**	
**Question**	**Response**
**Financial Disclosure**	The author(s) received no specific funding for this work.

Powered by Editorial Manager® and ProduXion Manager® from Aries Systems Corporation

Enter a financial disclosure statement that describes the sources of funding for the work included in this submission. Review the submission guidelines for detailed requirements. View published research articles from *PLOS ONE* for specific examples.<br><br>This statement is required for submission and **will appear in the published article** if the submission is accepted. Please make sure it is accurate.<br><br>**Unfunded studies**<br>Enter: *The author(s) received no specific funding for this work.*<br><br>**Funded studies**<br>Enter a statement with the following details:<br>• Initials of the authors who received each award<br>• Grant numbers awarded to each author<br>• The full name of each funder<br>• URL of each funder website<br>• Did the sponsors or funders play any role in the study design, data collection and analysis, decision to publish, or preparation of the manuscript?<br>• **NO** - Include this sentence at the end of your statement: *The funders had no role in study design, data collection and analysis, decision to publish, or preparation of the manuscript.*<br>• **YES** - Specify the role(s) played.<br><br>* typeset	
**Competing Interests**<br><br>Use the instructions below to enter a competing interest statement for this submission. On behalf of all authors, disclose any competing interests that could be perceived to bias this work—acknowledging all financial support and any other relevant financial or non-financial competing interests.<br><br>This statement is required for submission and **will appear in the published article** if	The authors have declared that no competing interests exist.

Powered by Editorial Manager® and ProduXion Manager® from Aries Systems Corporation

the submission is accepted. Please make sure it is accurate and that any funding sources listed in your Funding Information later in the submission form are also declared in your Financial Disclosure statement.<br><br>View published research articles from *PLOS ONE* for specific examples.<br><br>**NO authors have competing interests**<br><br>Enter: *The authors have declared that no competing interests exist.*<br><br>**Authors with competing interests**<br><br>Enter competing interest details beginning with this statement:<br><br>*I have read the journal's policy and the authors of this manuscript have the following competing interests: [insert competing interests here]*<br><br>* typeset	
**Ethics Statement**<br><br>Enter an ethics statement for this submission. This statement is required if the study involved:<br><br>• Human participants<br>• Human specimens or tissue<br>• Vertebrate animals or cephalopods<br>• Vertebrate embryos or tissues<br>• Field research<br><br>Write "N/A" if the submission does not require an ethics statement.<br><br>General guidance is provided below. Consult the submission guidelines for detailed instructions. **Make sure that all information entered here is included in the Methods section of the manuscript.**	N/A

**Format for specific study types**<br><br>**Human Subject Research (involving human participants and/or tissue)**<br>• Give the name of the institutional review board or ethics committee that approved the study<br>• Include the approval number and/or a statement indicating approval of this research<br>• Indicate the form of consent obtained (written/oral) or the reason that consent was not obtained (e.g. the data were analyzed anonymously)<br><br>**Animal Research (involving vertebrate animals, embryos or tissues)**<br>• Provide the name of the Institutional Animal Care and Use Committee (IACUC) or other relevant ethics board that reviewed the study protocol, and indicate whether they approved this research or granted a formal waiver of ethical approval<br>• Include an approval number if one was obtained<br>• If the study involved *non-human primates*, add *additional details* about animal welfare and steps taken to ameliorate suffering<br>• If anesthesia, euthanasia, or any kind of animal sacrifice is part of the study, include briefly which substances and/or methods were applied<br><br>**Field Research**<br><br>Include the following details if this study involves the collection of plant, animal, or other materials from a natural setting:<br>• Field permit number<br>• Name of the institution or relevant body that granted permission	
**Data Availability**<br><br>Authors are required to make all data underlying the findings described fully available, without restriction, and from the time of publication. PLOS allows rare exceptions to address legal and ethical concerns. See the PLOS Data Policy and FAQ for detailed information.	Yes - all data are fully available without restriction

A Data Availability Statement describing where the data can be found is required at submission. Your answers to this question constitute the Data Availability Statement and **will be published in the article**, if accepted.<br><br>**Important:** Stating 'data available on request from the author' is not sufficient. If your data are only available upon request, select 'No' for the first question and explain your exceptional situation in the text box.<br><br>Do the authors confirm that all data underlying the findings described in their manuscript are fully available without restriction?	
**Describe where the data may be found in full sentences. If you are copying our sample text, replace any instances of XXX with the appropriate details.**<br><br>• If the data are **held or will be held in a public repository**, include URLs, accession numbers or DOIs. If this information will only be available after acceptance, indicate this by ticking the box below. For example: *All XXX files are available from the XXX database (accession number(s) XXX, XXX.)*.<br>• If the data are all contained **within the manuscript and/or Supporting Information files**, enter the following: *All relevant data are within the manuscript and its Supporting Information files.*<br>• If neither of these applies but you are able to provide **details of access elsewhere**, with or without limitations, please do so. For example:<br><br>*Data cannot be shared publicly because of [XXX]. Data are available from the XXX Institutional Data Access / Ethics Committee (contact via XXX) for researchers who meet the criteria for access to confidential data.*<br><br>*The data underlying the results presented in the study are available from (include the name of the third party*	All relevant data are within the manuscript and its Supporting Information files.

and contact information or URL).<br>• This text is appropriate if the data are owned by a third party and authors do not have permission to share the data.<br><br>* typeset	
Additional data availability information:	

Manuscript Click here to access/download;Manuscript;Manuscript.docx

Effect of External Therapies of Traditional Chinese Medicine on Constipation in Patients with CRF: A Meta-Analysis

Yu Wu[1], Qisu Ying[1], Yajing He[1], Xiangcheng Xie[1], Xiao Yuan[2], Ming Wang[1], Xiao Fei[1], Xiu Yang[1*]

[1] Department of Nephrology, Affiliated Hangzhou First People's Hospital, School of Medicine, Zhejiang University, Hangzhou 310006, China.

[2] Department of Nursing, Affiliated Hangzhou First People's Hospital, School of Medicine, Zhejiang University, Hangzhou 310006, China.

*Correspondence should be addressed to Xiu Yang; yxyyxx@126.com.

ABSTRACT

Objective The purpose of this study was to evaluate the curative effect of external therapies of traditional Chinese medicine in chronic constipation and to provide scientific theoretical basis for clinical practice. ***Method*** We searched the English database of PubMed, EMBASE, the Cochrane Library and the Web of Science and Chinese database of CNKI, Wan fang database, VIP Database and China Biomedical Literature Database up to December 2022. Randomized controlled trials (RCTs) involving constipation in patients with CRF that compared external therapies of traditional Chinese medicine and routine treatment to routine treatment were eligible for the analysis. A meta-analysis of the outcome indicators including total efficiency, weekly defecation times, defecation time, defecation difficulty score, patient-assessment of constipation quality of life and adverse events of treatment were performed. The analysis was performed by using Review Manager version 5.3. ***Result***

A total of 23 studies were included，with 1764 patients. Meta-analysis results showed that compared with the control group , the test group could significantly increase weekly defecation times(MD = 0.94，95%CI（0.70，1.18），Z=7.74，$P < 0.00001$), reduce defecation time(MD = -2.92，95%CI（-3.69，-2.16），Z=7.49，$P < 0.00001$), reduce defecation difficulty score(MD = -1.92，95%CI（-2.45，-1.39），Z=7.11，$P < 0.00001$), improve the quality of life in patients with constipation(MD = -7.57，95%CI(-10.23，-4.91)，Z=5.58，$P < 0.00001$) and obtain a higher total effective rate of treatment(OR=4.53，95%CI（3.27，6.29），Z=9.07，$P < 0.00001$). In terms of safety, there was no significant difference in the incidence of adverse events between two groups(OR=0.36，95%CI（0.04，2.95），Z=0.95，P =0.34).***Conclusion*** The combination of external therapies of traditional Chinese medicine and routine treatment could achieve an excellent curative effect, and there was no specific adverse event. However because of the limited level of current evidence, more high-quality trials are needed in the future.

1. Introduction

Constipation is a common disease, It is reported that constipation is a symptom that affects 11-20% adult population every year[1].And the mean prevalence rate of chronic constipation is approximately 14% all over the world [2]. In China the prevalence rate in adults is 7.0%~20.3%[3].From the most recently published diagnosis criteria which is from Rome Committee in 2016 (Rome IV), The definition of constipation includes that straining, lumpy or hard stools, the sensation of incomplete evacuation, the sensation of anorectal obstruction or blockage, the use of manual manoeuvres to

facilitate defecation, and fewer than three bowel movements per week, any two or more items of these symptoms continued for at least 3 months in the preceding 6 months can qualify the patient for a diagnosis of functional constipation[4]. Constipation may occur because of a primary motor disorder involving the colon or can be induced by ~~kinds of~~ secondary causes, It may be associated with a large number of diseases or as an adverse effect of many drugs[5]. For example, uremia in CKD (chronic kidney disease) patients, electrolyte disorders like hypercalcemia, hypokalemia (severe) and hypothyroidism (severe) which are common symptoms in uremia are some of important causes of constipation [5]. And constipation is also a more common problem for patients receiving hemodialysis[6]. According to statistics, approximately 53% patients with end-stage renal disease who are receiving dialysis are suffering from constipation[7]. And the incidence of constipation is 71.7% in haemodialysis patients and 14.2% in peritoneal dialysis patients respectively[8].A global multicenter study showed that chronic constipation could significantly reduce the quality of life, as well as in China[9-10].The treatment of constipation includes general treatment of reasonable diet, plenty of water, moderate exercise and establishing good defecation habits, which are the basic treatment measures for patients with chronic constipation, and medical treatment includes the administration of stimulant and osmotic laxatives, new intestinal secretagogues and peripherally restricted μ-opiate receptor antagonists et al[5, 11]. Abusing of laxatives or repeated medical treatment can cause ~~bring~~ patients a heavy financial burden[12]. For the past few years, external therapies of traditional Chinese medicine had played a very important

role in the treatment for constipation in people diagnosed with chronic renal failure[13]. However, whether traditional Chinese external therapies are effective or superior to routine treatment measures remain to be clarified.

In order to further evaluate the curative effect of external therapies of traditional Chinese medicine in chronic constipation and to provide scientific theoretical basis for clinical practice, this study systematically evaluated and analyzed the clinical research literature of external therapies of traditional Chinese medicine on constipation in patients with chronic renal failure.

This meta-analysis was performed in accordance with the recommendations of the Cochrane handbook for systematic reviews of interventions and was reported in compliance with Meta-Analyses (PRISMA) statement guidelines.

2. Methods

2.1 Data sources and search strategy

We conducted a search of English database of PubMed, EMBASE, the Cochrane Library and the Web of Science and Chinese database of China National Knowledge Infrastructure (CNKI), Wan-Fang database, VIP Database and China Biomedical Literature Database (CBM) which were published between establishment of the database and December 2022 using the search terms *constipation, Renal Insufficiency, Chronic, Renal Dialysis, Acupuncture Therapy, Moxibustion, Acupoint sticking therapy, Tuina, Massage, Cupping Therapy, Auricular point sticking, Acupuncture, Ear, Enema, Acupoint Catgut-Embedding Therapy, placebo and RCTs.*

2.2 Inclusion and exclusion criteria

The inclusion criteria were developed by using a PICOS (Patient, Intervention, Comparators, Outcome, Study design) approach. The study subjects were adult patients who were diagnosed with CRF and constipation and there were specific diagnostic criteria for constipation in the study; the study compared routine treatment combined with different ways of external therapies of traditional Chinese medicine versus routine treatment and there was no limit to the course of treatment; all the studies were randomized controlled trials. The outcome indicators included total effective rate, weekly defecation times, defecation time, clinical constipation scores, PAC-QOL and occurrence of adverse events.

Studies were rejected according to the exclusion criteria as follows: constipation was caused by organic lesions of the intestinal tract(such as tumor, Crohn's disease, colonic polyp, intestinal tuberculosis et al), repeatedly published literature or data repeated in other articles or data included in other articles, no clear evaluation standard of curative effect, no data available for this study in the article, animal experiments, reviews, case reports, expert experience reports, conference abstracts and article not in Chinese or English.

2.3 Data extraction

Data from the retrieved literature were extracted and analyzed independently by two reviewers (WY and YYS), if there were different opinions about the quality of a study, a third reviewer examined the controversial literature and discussed it with the two aforementioned reviewers to resolve the discussion. And data were included only if the three reviewers achieved consensus regarding the data. The extracted data mainly

included the first author, publication year, sample size, age, sex, intervention, treatment time, outcome indications and so on.

2.4 Quality assessment

Two authors (WY and YYS) independently evaluated risk of methodological quality of the included literature by using the Cochrane risk-of-bias tool. There were seven items: random sequence generation, allocation concealment, blinding of participants and personnel, blinding of outcome assessment, incomplete outcome data, selective reporting, and other bias. The evaluation results of each item were divided into three levels: low-risk, unclear and high-risk, which were represented by a score of bias.

2.5 Statistical analyses

Analyses were performed using Review Manager 5.3. Dichotomous outcome data were measured with odds ratio (OR) and 95% CI, and continuous outcomes were measured with mean difference (MD) or standardized mean difference (SMD) and 95% CI, and we assessed changes based on mean values and standard deviations (SDs) changes between the pre-treatment and post-treatment. The heterogeneity between studies was evaluated by the chi-square-based Q statistical test using the heterogeneity χ^2 and I^2 statistics. In all analyses, $P < 0.05$ was deemed to represent significant heterogeneity, and the random-effects models were used for the meta-analysis of each indicator and vice versa. In addition, publication bias was assessed by using funnel plots.

3. Results

3.1 Searching Results

The search strategy generated 1409 citations, of which 79 published articles appeared to be relevant to the systematic review and were retrieved for further assessment. Of these studies, 56 were excluded. Overall we included 23 eligible articles reporting on 23 separate trials ultimately. These trails contained 1764 patients who were allocated to test group and control group. The search process was illustrated in Fig. 1, and detailed characteristics of the eligible studies were listed in Table 1.

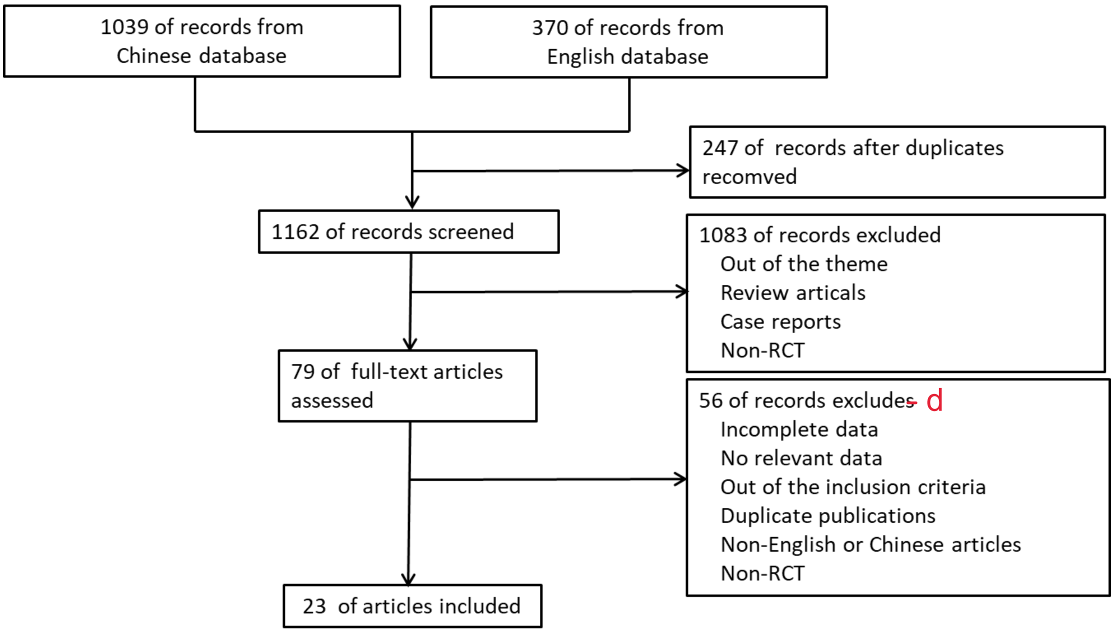

Figure 1 Flow diagram of literature search and selection of included studies.

Table 1 Characteristics of ~~trails~~ trials included in the study.

Study	Sample	Intervention(treatment)	Intervention (control)	Follow-up	Outcomes
Zhao 2015[14]	50（25/25）	Routine treatment + Seed-embedding at otopoints combined with abdominal massage	Routine treatment	4W	①②③
Shen 2015[15]	90(45/45)	Routine treatment + Auricular-plaster therapy	Routine treatment	4W	①
Li 2015[16]	67(34/33)	Routine treatment + moxibustion therapy	Routine treatment	8W	①
Zhang 2016[17]	60(30/30)	Routine treatment + massage + ultrasonic drug penetration therapy	Routine treatment + massage	1W	①②③
Wang 2016[18]	100(50/50)	Routine treatment + massage combined with Daihuang Ointment application on Shenque (CV 8)	Routine treatment	1W	①
Ma 2017[19]	50(25/25)	Routine treatment + moxibustion on abdominal acupoints	Routine treatment	4W	④
Chen 2018[20]	78(39/39)	Routine treatment +Seed-embedding at otopoints combined with abdominal massage	Routine treatment	/	①
Zhang 2018[21]	68(34/34)	Routine treatment + auricular pressure	Routine treatment	8W	①⑤
Li 2018[22]	80(40/40)	Routine treatment +Seed-embedding at otopoints combined with abdominal massage	Routine treatment	4W	②③
Li 2018[23]	90(45/45)	Routine treatment + Auricular embedding beans combined with abdominal massage	Routine treatment	/	①③
Huang 2019[24]	96(48/48)	Routine treatment +Seed-embedding at otopoints combined with moxibustion therapy	Routine treatment	1W	①④
Li 2019[25]	60(30/30)	Routine treatment + Acupressure combined with abdominal massage	Routine treatment	4W	①
Ding 2019[26]	60（30/30）	Routine treatment + Umbilical cord steaming therapy	Routine treatment	4W	①
Abbasi 2019[27]	70(35/35)	Routine treatment + Acupressure	Routine treatment	4W	②
Lu 2020[28]	84(42/42)	Routine treatment +Seed-embedding at otopoints combined with moxibustion therapy	Routine treatment	1W	①④
Lou 2020[29]	120(60/60)	Routine treatment + Acupoint-sticking therapy	Routine treatment	4W	②⑥

Study	Sample	Intervention(treatment)	Intervention (control)	Follow-up	Outcomes
Wang 2021[30]	120(60/60)	Routine treatment + Seed-embedding at Otopoints combined with acupoint-sticking therapy	Routine treatment	4W	①⑥
Zhang 2021[31]	68(34/34)	Routine treatment +Seed-embedding at otopoints combined with acupressure	Routine treatment	4W	②③
Fu 2021[32]	50(25/25)	Routine treatment + Acupoint Embedding at otopoints	Routine treatment	4W	⑤
Yan[33] 2021	80(40/40)	Routine treatment + Chinese medicine acupoint application	Routine treatment	4W	①⑥
Li[34] 2022	83(42/41)	Routine treatment + Auricular point pressing combined with Umbilical sticking	Routine treatment	4W	②③⑥
Li[35] 2022	50(25/25)	Routine treatment + Acupoint Embedding at otopoints	Routine treatment	48W	⑤
Chen[36] 2022	90(45/45)	Routine treatment +Acupuncture therapy + Chinese medicine acupoint application	Routine treatment	12W	⑥

①total efficiency; ②Weekly defecation times; ③Defecation time; ④Defecation difficulty score; ⑤Adverse events of treatment; ⑥Patient-Assessment of Constipation Quality Of Life(PAC-QOL)

3.2 Quality of trials

Of the 23 eligible randomized controlled trials, 18 studies described the grouping method as the random number table method, 4 studies only mentioned "random" and 1 study was numbered according to the order of hospital admission and then grouped according to the random number table method. And only 2 studies were double blinded trials. None of the studies reported the reporting bias and the existence of other biases(Fig 2).

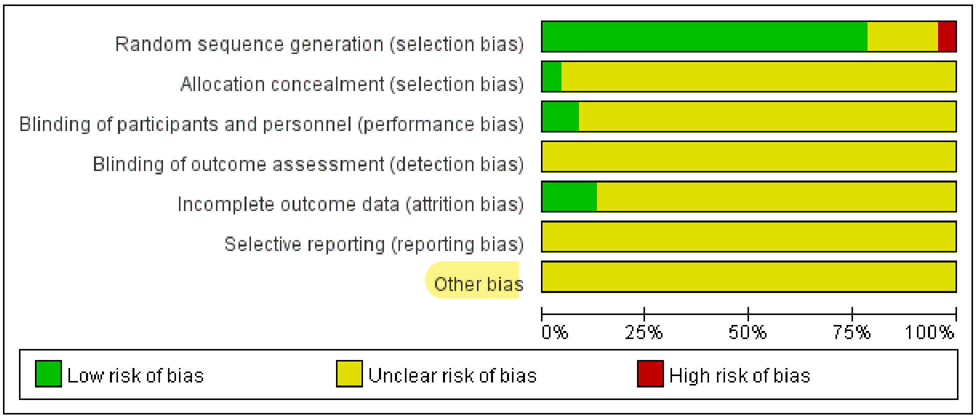

Fig. 2 Risk of bias graph of included studies

3.3 Outcome Analysis

3.3.1 Total efficiency. 14 studies evaluated the effectiveness of the treatment of which 552 patients were assigned to treatment group and 551 were allocated to control group. 8 studies [14, 16, 17, 20, 21, 23, 25,33] evaluated the therapeutic effect according to the standard of "Guidance Principle of clinical study on new drug of traditional Chinese medicine [14]". Efficacy? Effectivity means that the time between defecation is shortened by 1 day, and all other symptoms are improved. The failure? invalidation of treatment means that constipation symptoms do not improve or increase. And 6 studies[15, 18, 24, 26, 28, 30] evaluated the therapeutic effect according to "Standard for traditional Chinese

efficacy?

medicine clinical diagnosis and efficacy[30]". The effectivity means that the patient can defecate once within 3 days, and the clinical symptoms are relieved, the failure? invalidation of treatment means that constipation symptoms do not improve or increase. Total effective rate = (number of effective cases/total cases) × 100%.

There was no statistical heterogeneity among the studies (I^2 = 0%, P = 0.87), the fixed effect model was used for analysis. The results showed that there did have significantly statistical differences in total efficacy or efficiency? efficiency between treatment and control group〔OR=4.53，95%CI（3.27，6.29），Z=9.07，P＜0.00001;Fig 3〕. meaning not clear

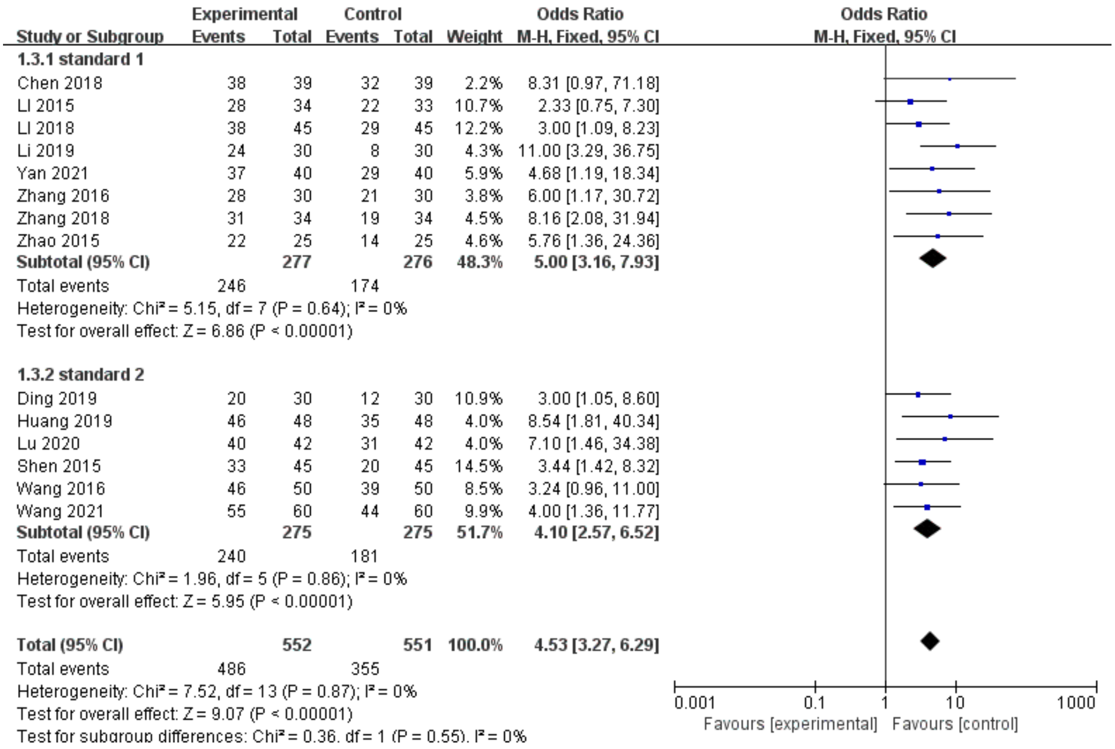

Fig. 3 Forest plot of total efficiency in the treatment group and the control group.

3.3.2 Weekly defecation times. 7 studies[14, 17, 22, 27, 29, 31,34] evaluated the weekly defecation times after the treatment of which 266 patients were assigned to treatment group and 265 patients were assigned to control group respectively. There ~~had~~ was statistical heterogeneity among the studies (I^2 = 72%, P = 0.002), the random effect

model was used for analysis. The results showed that there ~~did have~~ was significant~~ly~~ statistical differences in weekly defecation times changes between baseline and end of treatment between treatment and control group（MD = 0.94，95%CI（0.70，1.18），Z=7.74，P < 0.00001;Fig 4）.

Study or Subgroup	Experimental Mean	Experimental SD	Experimental Total	Control Mean	Control SD	Control Total	Weight	Mean Difference IV, Random, 95% CI
Abbasi 2019	1.7	1.56	35	0.36	1.36	35	7.9%	1.34 [0.65, 2.03]
Li 2018	1.24	0.99	40	0.5	0.85	40	13.8%	0.74 [0.34, 1.14]
Li 2022	2.15	0.87	42	1.05	0.55	41	16.4%	1.10 [0.79, 1.41]
Lou 2020	2.13	0.32	60	1.42	0.22	60	22.0%	0.71 [0.61, 0.81]
Zhang 2016	1.77	0.65	30	0.48	0.7	30	15.6%	1.29 [0.95, 1.63]
Zhang 2021	1.55	0.94	34	0.42	0.9	34	12.9%	1.13 [0.69, 1.57]
Zhao 2015	0.92	0.97	25	0.48	0.86	25	11.2%	0.44 [-0.07, 0.95]
**Total (95% CI)**			**266**			**265**	**100.0%**	**0.94 [0.70, 1.18]**

Heterogeneity: Tau² = 0.06; Chi² = 21.22, df = 6 (P = 0.002); I² = 72%
Test for overall effect: Z = 7.74 (P < 0.00001)

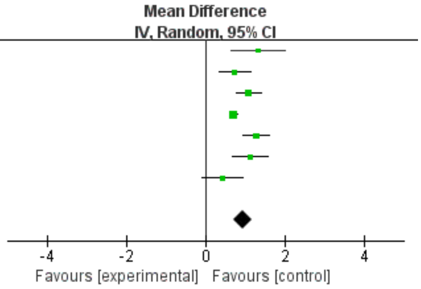

Fig. 4 Forest plot of weekly defecation times changes between baseline and end of treatment between treatment and control group.

3.3.3 Defecation time. 6 studies[14, 17, 22, 23, 31,34] evaluated the defecation time after the treatment of which 216 patients were assigned to treatment group and 215 patients were assigned to control group respectively. There ~~had~~ was no statistical heterogeneity among the studies (I^2 = 0%, P = 0.73), the fixed effect model was used for analysis. The results showed that there ~~did have~~ was significant~~ly~~ statistical differences in defecation time changes between baseline and end of treatment between treatment and control group（MD = -2.92，95%CI（-3.69，-2.16），Z=7.49，P < 0.00001 Fig5）.

Study or Subgroup	Experimental Mean	Experimental SD	Experimental Total	Control Mean	Control SD	Control Total	Weight	Mean Difference IV, Fixed, 95% CI
Li 2018	-5.75	4.7	40	-3.47	7.97	40	7.1%	-2.28 [-5.15, 0.59]
LI 2018	-11.6	3.72	45	-8.7	5.42	45	15.8%	-2.90 [-4.82, -0.98]
Li 2022	-7.33	3.11	42	-5.24	3.87	41	25.6%	-2.09 [-3.60, -0.58]
Zhang 2016	-14.33	11.47	30	-9.12	8.74	30	2.2%	-5.21 [-10.37, -0.05]
Zhang 2021	-5.86	2.43	34	-2.39	3.13	34	33.0%	-3.47 [-4.80, -2.14]
Zhao 2015	-4.36	3.28	25	-1.24	3.54	25	16.3%	-3.12 [-5.01, -1.23]
**Total (95% CI)**			**216**			**215**	**100.0%**	**-2.92 [-3.69, -2.16]**

Heterogeneity: Chi² = 2.80, df = 5 (P = 0.73); I² = 0%
Test for overall effect: Z = 7.49 (P < 0.00001)

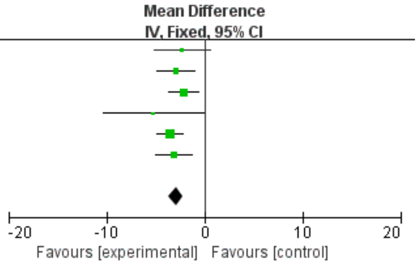

Fig. 5 Forest plot of defecation time changes between baseline and end of treatment in the treatment group and the control group.

3.3.4 Defecation difficulty score. 3 studies[19, 24, 28] compared the defecation difficulty score after the treatment of which 115 patients were assigned to treatment group and control group respectively. There had statistical heterogeneity among the studies (I^2 = 63%, P = 0.07), the random effect model was used for analysis. The results showed that there did have significantly statistical differences in defecation difficulty score changes between baseline and end of treatment between treatment and control group（MD = -1.92，95%CI（-2.45，-1.39），Z=7.11，P＜0.00001; Fig 6）.

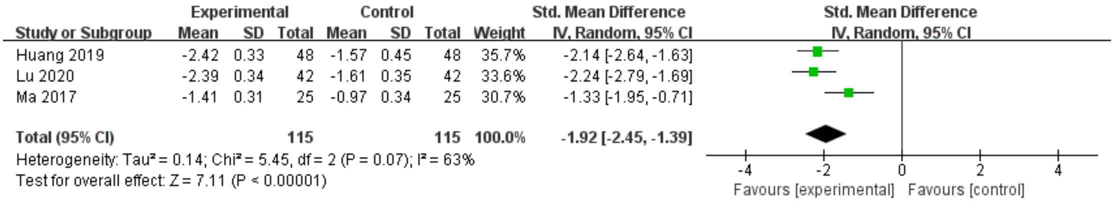

Fig. 6 Forest plot of defecation difficulty score changes between baseline and end of treatment in the treatment group and the control group.

3.3.5 Patient-Assessment of Constipation Quality Of Life (PAC-QOL). 5 studies [29, 30, 33,34,36] compared the quality of life in patient with constipation after the treatment of which 247 patients were assigned to treatment group and 246 patients were assigned to control group respectively. There had statistical heterogeneity among the studies (I^2 = 64%, P = 0.03), the random effect model was used for analysis. The results showed that there did have significantly statistical differences in quality of life in patients between baseline and end of treatment between treatment and control group（MD = -7.57，95%CI（-10.23，-4.91），Z=5.58，P＜0.00001; Fig 7）.

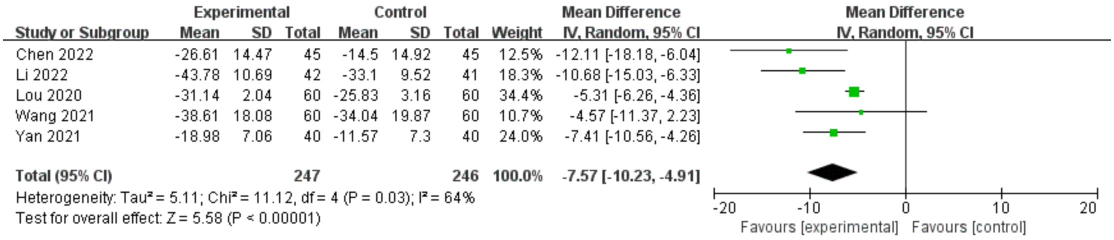

Fig. 7 Forest plot of PAC-QOL changes between baseline and end of treatment in the treatment group and the control group.

3.3.6 Adverse events of treatment. 3 studies[21, 32,35] reported the occurrence of adverse events during treatment, including intestinal obstruction, hematochezia and allergic reactions characterized by skin redness and itching after auricular point sticking. There ~~had~~ was statistical heterogeneity among the studies (I^2 = 55%, P = 0.11), the random effect model was used for analysis. The results showed that there ~~had~~ was no significant~~ly~~ statistical differences in the incidence of adverse events between treatment and control group〔OR=0.36，95%CI（0.04，2.95），Z=0.95，P =0.34; Fig **8**）.

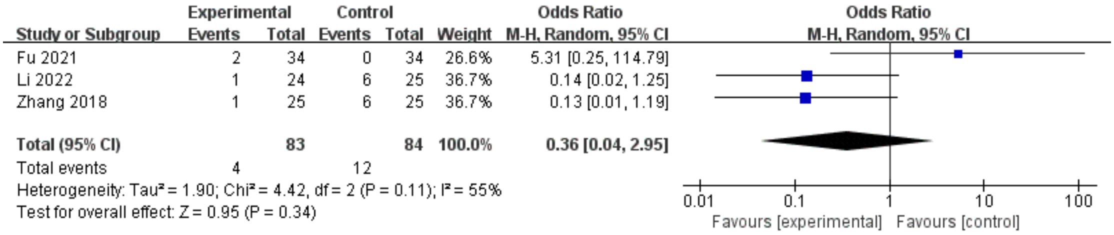

Fig. 8 Forest plot of adverse events in the treatment group and the control group.

3.4 Funnel Plot of Publication Bias

As shown in Figure 9, a funnel chart analysis of clinical effectiveness rate was performed. Results showed that the scatter is basically distributed at the top of the funnel chart which indicated that the risk of publication bias in the clinical efficacy of constipation is low.

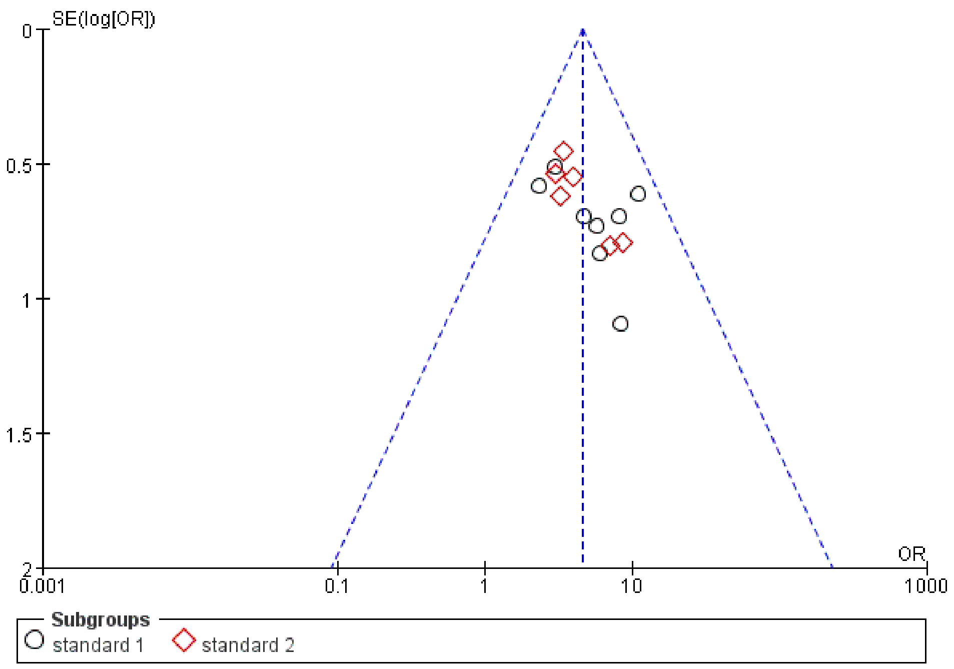

Fig. 9 Funnel plot of effectiveness rate in the treatment group and the control group.

4. Discussion

Constipation is a ~~prevalent~~ disorder with a global prevalence of 14%[2]. It's prevalence increases with age and is almost twice as common in women than men[37], which can significantly interfere with patients' daily life style ~~living~~ and sense of well-being and consumes resources in healthcare systems worldwide[38-40]. The prevalence of constipation in dialysis patients is reported to be 36.3%~66.7%[41] and has been shown to be higher in patients with CKD than the general population, particularly among those undergoing dialysis[42,43]. Water restriction, water removal by dialysis, inadequate intake of dietary fiber due to potassium restriction and the associated changes in intestinal microflora, lack of exercise, diabetic autonomic nervous system disorder, intake of potassium inhibitors and phosphorus adsorbents may be the reasons why dialysis patients are prone to develop constipation[44,45].However, A considerable proportion of constipated people were reported dissatisfied with conventional treatments, because of the lack of

effectiveness and the side effects[46-49].

In recent years, several studies had shown that external therapies of traditional Chinese medicine such as acupuncture, moxibustion, massage, cupping therapy, ~~and~~ ear acupunctures and so on were effective in the treatment of constipation, and compared with conventional treatments, they were more effective, non-toxic and without side effects, low cost, more convenient operation and suitable for long-term medication[50-54]. And in this meta-analysis, the therapeutic effect of external therapy of traditional Chinese medicine on chronic renal failure patients with constipation was explored. Our results suggested that external therapy of traditional Chinese medicine can significantly increase the frequency of weekly defecation, shorten the defecation time, and obtain a higher total effective rate of treatment. And there were no obvious adverse events in the course of treatment.

A meta-analysis [55] in 2022 showed that treatment with Seed-embedding at otopoints plus routine treatment had a~~n~~ better effect on dialysis patients with constipation compared to treatment with routine treatment alone. However, that study only discussed the external therapy of traditional Chinese medicine, which was based on Seed-embedding at otopoints and that study only included patients with maintenance hemodialysis. Our meta-analysis covers a wider range of subjects and treatments.

The meta-analysis had some limitations. First, Some studies did not describe the randomization process or the procedure of allocation concealment in detail; Second, the sample size was small in some studies. Third, most of the studies in this meta-analysis were Chinese-based, which may have caused regional, language, and

racial biases. We await further well-designed and high-quality RCTs to further provide reliable data.

5. Conclusion

In conclusion, External therapies of traditional Chinese medicine are safe and effective ways to relieve symptoms of constipation in people with CRF.

Conflicts of Interest

The authors declare that there are no conflicts of interest regarding the publication of this paper.

Authors' Contributions

Yu Wu and Xiu Yang designed the study. Yu Wu and Qishu Ying checked the literature. Yu Wu, Qishu Ying, Yajin He analyzed the data. Xiu Yang supervised the study. Yu Wu wrote the manuscript. Yu Wu and Qishu Ying critically revised the manuscript for important intellectual content.

Acknowledgements

This study was supported by Grants from Zhejiang Provincial Traditional Chinese Medicine Science and Technology Project (2021ZB223).

References

[1] Schiller L R. Chronic constipation: new insights, better outcomes?[J]. Lancet Gastroenterol Hepatol,2019,4(11):873-882.DOI：10.1016/S2468-1253(19)30199-2.

[2] Suares N C, Ford A C. Prevalence of, and risk factors for, chronic idiopathic constipation in the community: systematic review and meta-analysis[J]. Am J Gastroenterol,2011,106(9):1582-1591, 1581, 1592.DOI：10.1038/ajg.2011.164. Epub 2011 May 24.

[3] Long Y, Huang Z, Deng Y, et al. Prevalence and risk factors for functional bowel disorders in South China: a population based study using the Rome III criteria[J]. Neurogastroenterol Motil,2017,29(1).DOI：10.1111/nmo.12897. Epub 2016 Jul 14.

[4] Mearin F, Lacy B E, Chang L, et al. Bowel Disorders[J]. Gastroenterology,2016.DOI：10.1053/j.gastro.2016.02.031.

[5] Wald A. Constipation: Advances in Diagnosis and Treatment[J]. JAMA,2016,315(2):185-191.DOI: 10.1001/jama.2015.16994.

[6] Wu M J, Chang C S, Cheng C H, et al. Colonic transit time in long-term dialysis patients[J]. Am J Kidney Dis,2004,44(2):322-327.DOI: 10.1053/j.ajkd.2004.04.048.

[7] Murtagh F E, Addington-Hall J, Higginson I J. The prevalence of symptoms in end-stage renal disease: a systematic review[J]. Adv Chronic Kidney Dis,2007,14(1):82-99.DOI: 10.1053/j.ackd.2006.10.001.

[8] Zhang J, Huang C, Li Y, et al. Health-related quality of life in dialysis patients with constipation: a cross-sectional study[J]. Patient Prefer Adherence,2013,7:589-594.DOI: 10.2147/PPA.S45471. Print 2013.

[9] Wald A, Scarpignato C, Kamm M A, et al. The burden of constipation on quality of life: results of a multinational survey[J]. Aliment Pharmacol Ther,2007,26(2):227-236.DOI: 10.1111/j.1365-2036.2007.03376.x.

[10]Xiong L S, Chen M H, Chen H X, et al. A community population-based epidemiologic study of chronic constipation in Guangdong province[J].Chinese Journal of Digestion, 2004,24:488-491.DOI: 10.3760/j.issn:0254-1432.2004.08.011.

[11]Chinese Society of gastroenterology, Chinese Society of Colorectal Surgery.Guidelines for the

diagnosis and treatment of chronic constipation in China(2013, WuHan)[J].Chinese Journal of Gastroenterology, 2013,18(10):605-612.DOI: 10.3969/j.issn.1008-7125.2013.10.007.

[12]Mohaghegh S H, Soori H, Khoshkrood M B, et al. Direct and indirect medical costs of functional constipation: a population-based study[J]. Int J Colorectal Dis,2011,26(4):515-522.

[13] Li D N, Zhang L, Zheng X, et al. Research Progress in the Treatment of Chronic Renal Failure Combined with Constipation by Traditional Chinese and Western Medicine[J]. Henan Traditional Chinese Medicine, 2020,40(07):1113-1117.DOI: 10.16367/j.issn.1003-5028.2020.07.0282.

[14]Zhao Y H, Tian C Y, Rong J Y, et al. The Clinical Nursing of Seed-embedding at Otopoints Combined with Abdominal Massage to Improve Maintenance Hemodialysis Patients with Constipation[J]. Chinese Medicine Modern Distance Education of China, 2015,13: 125-126.DOI: 10.3969/j.issn.1672-2779.2015.05.060.

[15]Shen J J, Wang X D, Zhang Y L. Effect of Auricular-plaster Therapy on Constipation in Patients with Maintenance Hemodialysis[J]. Journal of Nursing, 2015:62-64.DOI: 10.16460/j.issn1008-9969.2015.01.062.

[16]Li N, Xiao X R, Duan Y F, et al. Clinical observation on treating chronic constipation of patients of hemodialysis with deficiency syndrome by using moxibustion therapy[J]. China Journal of Traditional Chinese Medicine and Pharmacy, 2015,30:1331-1333.

[17]Zhang X P. Effect of ultrasonic drug penetration therapy on Constipation in Patients with Maintenance Hemodialysis [J]. Medical Journal of the Chinese People's Armed Police Forces, 2016,27:1264-1265. 10.3969/j.issn.1004-3594.2016.12.025.

[18]Wang F J, Huang Y L, Zheng H F. Clinical effect observation of massage combined with Daihuang Ointment application on Shenque (CV 8) on Constipation in Patients with chronic renal

failure[C].HangZhou, 2016:238-241.

[19]Ma W Z, Su Z W, Qu Z H. Clinical study of moxibustion on abdominal acupoints in the treatment of chronic kidney disease combined with functional constipation[J]. China Medical Herald, 2017,14:100-103.

[20]Chen M F, Li Y Q. The improvement effect of seed-embedding at otopoints combined with abdominal massage in Patients with Maintenance Hemodialysis[J]. Chinese Journal of Rural Medicine and Pharmacy, 2018,25:25-26. 10.3969/j.issn.1006-5180.2018.23.014.

[21]Zhang J J, Zhuang J, Shen C Z. Research on the effect of auricular pressure on uremic hemodialysis patients with asthenia type constipation[J].Nursing and Rehabilitation Journal, 2018,17:70-73.DOI: 10.3969/j.issn.1671-9875.2018.09.022.

[22]Li M J. The study of improvement effect of seed-embedding at otopoints combined with abdominal massage in Patients with Maintenance Hemodialysis[J]. Chinese Manipulation & Rehabilitation Medicine, 2018,9:29-30.DOI: 10.3969/j.issn.1008-1879.2018.16.013.

[23]Li Y Y, Han L, Wu J H, et al. The clinical nursing effect of auricular embedding beans combined with abdominal massage to improve constipation in maintenance hemodialysis patients[J].Journal of Colorectal & Anal Surgery, 2018:179-180. DOI: 10.19668/j.cnki.issn1674-0491.2018.S2.179.

[24]Huang L. Application of seed-embedding at otopoints combined with moxibustion therapy in Patients with Maintenance Hemodialysis[J].Chinese General Practice Nursing, 2019,17:1480-1482.DOI: 10.12104/j.issn.1674-4748.2019.12.028.

[25]Li Y W, Li J H, Cao Y S, Clinical effect observation on acupressure combined with abdominal massage in Patients with Maintenance Hemodialysis[J]. Beijing Journal of Traditional Chinese Medicine, 2019,38:606-608.DOI: 10.16025/j.1674-1307.2019.06.026.

[26]Ding L, Dai S S, Zhao J, et al. Clinical observation on umbilical cord steaming therapy in the treatment of constipation in the patients with IgA nephropathy undergoing maintenance hemodialysis[J]. China Modern Doctor, 2019,57:111-114, 118.

[27] Abbasi P, Mojalli M, Kianmehr M, et al. Effect of acupressure on constipation in patients undergoing hemodialysis: A randomized double-blind controlled clinical trial[J]. Avicenna Journal of Phytomedicine,2019,9(1):84-91.

[28]Lu A X. Application of Auricular Point Pressing with Beans Combined with Moxibustion for Maintenance Hemodialysis Complicated with Constipation[J]. Journal of New Chinese Medicine, 2020,52:178-180.DOI: 10.13457/j.cnki.jncm.2020.18.052.

[29]Lou L L, Cai H F, Yang L L, Clinical Study on Compound Congrong Dahuang Tongbian Patch Combined with Routine Medical Measures for Hemodialysis Complicated with Constipation[J]. Journal of New Chinese Medicine, 2020,52:141-144.DOI: 10.13457/j.cnki.jncm.2020.22.040.

[30]Wang L, Sun B L, Bao J L, et al. Clinical Observation on Auricular Point Sticking-Pressure Combined Shenque Point（CV8）Application in Treating Constipation in Elderly Patients with Type 2 Diabetic Nephropathy[J]. Journal of Guangzhou University of Traditional Chinese Medicine, 2021,38(1):85-90.DOI: 10. 13359/j. cnki. gzxbtcm. 2021. 01. 017.

[31]Zhang Y, Xiao H. Application of Acupoint Embedding Combined with Acupoint Pressing in Hemodialysis Patients with Constipation[J]. Chinese Journal of Dialysis and Artificial Organs, 2021,32(02):60-61.DOI: 10.3969/j.issn.1005-0809.2021.02.025.

[32]Fu J R, Li Y L, Wu S F. effect of acupoint embedding on constipation in elderly patients undergoing hemodialysis[J]. Shenzhen Journal of Integrated Traditional Chinese and Western Medicine, 2021,31(10):71-73.DOI: 10.16458/j.cnki.1007-0893.2021.10.032.

[33]Yan T T. Analysis of the Clinical Effect of Chinese Medicine Acupoint Application Combined with Hemodialysis Room Nursing Intervention on Constipation in Hemodialysis Patients[J]. China Foreign Medical Treatment, 2021,40(36): 13-16.DOI: 10.16662/j.cnki.1674-0742.2021.36.013.

[34]Li X H. Effect of Auricular Point Pressing Combined with Umbilical Sticking Nursing on Constipation in Patients with Maintenance Hemodialysis[J]. Guangming Journal of Chinese Medicine, 2022,37(17): 3174-3177.DOI: 10.3969 /j.issn.1003-8914.2022.17. 034.

[35]Li H A. Analysis of the effect of Auricular point sticking in elderly patients with hemodialysis constipation [J].XinJiang Journal of Traditional Chinese Medicine, 2022,40(01): 58-60.

[36]Chen R R, Liu Z H, Shao S S, et al. Acupuncture therapy combined with rhubarb applied to the umbilicus for constipation inpatients with stage IV-V diabetic nephropathy[J]. Chinese Journal of Primary Medicine and Pharmacy, 2022,29(2): 221-224.DOI: 10.3760/cma.j.issn.1008-6706.2022.02.014.

[37] Baffy N, Foxx-Orenstein A E, Harris L A, et al. Intractable Constipation in the Elderly[J]. Curr Treat Options Gastroenterol,2017,15(3):363-381.DOI: 10.1007/s11938-017-0142-2.

[38] Corsetti M, Brown S, Chiarioni G, et al. Chronic constipation in adults: Contemporary perspectives and clinical challenges. 2: Conservative, behavioural, medical and surgical treatment[J]. Neurogastroenterol Motil,2021,33(7):e14070.DOI: 10.1111/nmo.14070.

[39] Scott S M, Simrén M, Farmer A D, et al. Chronic constipation in adults: Contemporary perspectives and clinical challenges. 1: Epidemiology, diagnosis, clinical associations, pathophysiology and investigation[J]. Neurogastroenterol Motil,2021,33(6):e14050.DOI: 10.1111/nmo.14050.

[40] Sanchez M I, Bercik P. Epidemiology and burden of chronic constipation[J]. Can J

Gastroenterol,2011,25 Suppl B(Suppl B):11B-15B.DOI: 10.1155/2011/974573.

[41] Zuvela J, Trimingham C, Le Leu R, et al. Gastrointestinal symptoms in patients receiving dialysis: A systematic review[J]. Nephrology (Carlton),2018,23(8):718-727.DOI: 10.1111/nep.13243.

[42] Sumida K, Molnar M Z, Potukuchi P K, et al. Constipation and Incident CKD[J]. J Am Soc Nephrol,2017,28(4):1248-1258.DOI: 10.1681/ASN.2016060656.

[43] Shirazian S, Radhakrishnan J. Gastrointestinal disorders and renal failure: exploring the connection[J]. Nat Rev Nephrol,2010,6(8):480-492.DOI: 10.1038/nrneph.2010.84.

[44] Kamei D, Kamei Y, Nagano M, et al. Elobixibat alleviates chronic constipation in hemodialysis patients: a questionnaire-based study[J]. BMC Gastroenterol,2020,20(1):26.DOI: 10.1186/s12876-020-1179-6.

[45] Cano A E, Neil A K, Kang J Y, et al. Gastrointestinal symptoms in patients with end-stage renal disease undergoing treatment by hemodialysis or peritoneal dialysis[J]. Am J Gastroenterol,2007,102(9):1990-1997.DOI: 1111/j.1572-0241.2007.01321.x.

[46] Johanson J F, Kralstein J. Chronic constipation: a survey of the patient perspective[J]. Aliment Pharmacol Ther,2007,25(5):599-608.DOI: 10.1111/j.1365-2036.2006.03238.x.

[47] Sun S X, Dibonaventura M, Purayidathil F W, et al. Impact of chronic constipation on health-related quality of life, work productivity, and healthcare resource use: an analysis of the National Health and Wellness Survey[J]. Dig Dis Sci,2011,56(9):2688-2695.DOI: 10.1007/s10620-011-1639-5.

[48] Emmanuel A, Quigley E M, Simrén M, et al. Factors affecting satisfaction with treatment in European women with chronic constipation: An internet survey[J]. United European Gastroenterol J,2013,1(5):375-384.DOI: 10.1177/2050640613494200.

[49] Wainwright M, Russell A J, Yiannakou Y. Challenging the biopsychosocial model in a chronic constipation clinic[J]. Qual Health Res,2011,21(12):1643-1657.DOI: 10.1177/1049732311416824.

[50]Hu S Q, Zhao X Y, Zhao G Q, et al. Meta-analysis of Effectiveness of Traditional Chinese Medicine External Therapy for Treatment of Constipation in the Elderly[J]. Journal of Guangzhou University of Traditional Chinese Medicine, 2021,38(06):1198-1207.DOI: 10.13359/j.cnki.gzxbtcm.2021.06.022.

[51]Li D N, Zhang L, Zheng X, et al. Research Progress in the Treatment of Chronic Renal Failure Combined with Constipation by Traditional Chinese and Western Medicine[J]. Henan Traditional Chinese Medicine, 2020,40(07):1113-1117.DOI: 10.16367/j.issn.1003-5028.2020.07.0282.

[52]Liu F L, Liu B. Research Progress of Chronic Constipation Treated with external therapies of traditional Chinese medicine[J]. Chinese Journal of Coloproctology, 2019,39(12):75-76.DOI: 10.3969/j.issn.1000-1174.2019.12.039.

[53]Luo F, Wang Y P, Zhu Y. Research Progress of Chinese Medicine External Treatment on Functional Constipation[J]. Guiding Journal of Traditional Chinese Medicine and Pharmacology, 2017,23(02):86-88.

[54]Chinese Society of Gastroenterology , Neurogastroenterology and Motility Committee.Guidelines for the diagnosis and treatment of chronic constipation in China(2019, GuangZhou)[J]. Chinese Journal of Gastroenterology, 2019,39 (9): 577-598. DOI: 10.3760/cma.j.issn.0254-1432.2019.09.001.

[55]Jiang S F, Yang Z Z, Xue L Y. Improving constipation in hemodialysis patients with with Seed-embedding at otopoints: A meta-analysis[J]. Chinese Journal of Integrated Traditional and Western Nephrology, 2022,23(05):440-444. DOI:10.3969/j.issn.1009-587X.2022.05.019.

Click here to access/download
Supporting Information
PRISMA Checklist.docx
