# Supplementary material for: Effect of external therapies of traditional Chinese medicine on constipation in patients with CRF: A meta-analysis
Source: PLoS One. 2023 Oct 5;18(10):e0291968. doi: 10.1371/journal.pone.0291968 (PMC10553223; doi:10.1371/journal.pone.0291968)
Supplement: Attachment — Submitted filename: Reviewer comments_PLOS_ON20230402.docx [file pone.0291968.s003.docx]

**Reviewer’s comments and suggestions**

- **Major issues**

1. **The grammar for academic writing should be provided in this manuscript.**
2. **Please clarify the meaning of “CRF” in this work.**
3. **Introduction**
   3.1 **Please give information about CRF**
   3.2 **The importance of Chinese medicine practices for supportive treatment of constipation should additionally review**
4. **Methods**
   4.1 **Please give the rationale for selecting Chinese biomedical databases in this work because there are differences in utility and accessibility.**
   4.2 **In search terms, “renal insufficiency” and “Renal dialysis” seem not related to external therapy of conscription. Please clarify (minor issue).**
5. **Results**
   5.1 **Figure1 there is typing error “recomved”**
   5.2 **Table 1, Three pieces of research with too small sample sizes were analyzed in the work**

**(Zhao 2015, Fu 2021, Li 2022). The researcher should be concerned that too small a sample size leads to a lack of power in the study. It means that some outcomes have minor clinical interests or effects.**

5.3 **As reviewer understand in Figure 9 X-axis should be OR [LOG] and Y-axis should be standard error. If reviewer misunderstand, please clarify the results.**

6. **Discussion and conclusion**
   6.1 **Main point of each outcome indicator after analyzing should be discussed.**
   6.2 **Conclusion demonstrates overclaim results.**
