# Supplementary material for: Effect of external therapies of traditional Chinese medicine on constipation in patients with CRF: A meta-analysis
Source: PLoS One. 2023 Oct 5;18(10):e0291968. doi: 10.1371/journal.pone.0291968 (PMC10553223; doi:10.1371/journal.pone.0291968)
Supplement: Attachment — Submitted filename: Response to Reviewers.docx [file pone.0291968.s005.docx]

Reviewer’s comments and suggestions

	Major issues

**1. Please clarify the meaning of “CRF” in this work.**

Chronic renal failure

2.	Introduction

**2.1	Please give information about CRF**

Chronic renal failure (CRF) is the outcome of the continuous progression of various chronic kidney diseases, with metabolite retention and imbalance in the water, electrolytes, and acid base being the main manifestations of CRF

**2.2	The importance of Chinese medicine practices for supportive treatment of constipation should additionally review**

There are various ways of external therapies of traditional Chinese medicine of constipation, including acupuncture, moxibustion, massage, cupping therapy and so on. A large number of clinical studies had shown that the external therapies of traditional Chinese medicine was not only effective, but also could it avoid some adverse consequences such as abdominal pain, electrolyte disturbance, melanosis coli and severe drug dependence after long-term use of laxatives. For instance, the research of Zhang etal confirmed the exact effect and advantages of acupuncture in the treatment of functional constipation and by comparing massage with lactulose oral liquid in the treatment of constipation, Gao etal showed that tuina was more effective and there was no obvious adverse reaction, the research of Wang etalconfirmed that acupoint sticking therapy had unique advantages in the treatment of functional constipation because of simple operation, safe and non-invasive, satisfactory curative effect, low cost. The research of Bai etal confirmed the effect of cupping therapy on constipation and Luo etal confirmed the curative effect of ear-acupressure on constipation through systematic review.

3.	Methods

**3.1	Please give the rationale for selecting Chinese biomedical databases in this work because there are differences in utility and accessibility.**

Chinese Biomedical Literature Database is a comprehensive Chinese medical literature database, which contains more than 1600 Chinese biomedical journals since 1978, as well as the literature records of compilation and conference papers, with an annual increase of more than 400,000 articles and updated every month. CBM is the preferred database for systematic reviewers to retrieve relevant studies in Chinese(Ai C L, Duan Y R, WIFFEN PHIL, etal. Chinese Biomedical Databases: Selection and Search for Trials to Conduct Systematic Reviews [J]. Chinese Journal of Evidence-Based Medicine, 2010,10(06): 749-753.). We can go to the CBM database interface by retrieving http://www.sinomed.ac.cn/.

**3.2	In search terms, “renal insufficiency” and “Renal dialysis” seem not related to external therapy of conscription. Please clarify (minor issue).**

In this study, we evaluated the curative effect of external therapies of traditional Chinese medicine on constipation in patients with chronic renal failure, so we searched the terms of “renal insufficiency” and “Renal dialysis”.

4.	Results

**4.1	Figure1 there is typing error “recomved”**

removed

**4.2	Table 1, Three pieces of research with too small sample sizes were analyzed in the work(Zhao 2015, Fu 2021, Li 2022). The researcher should be concerned that too small a sample size leads to a lack of power in the study. It means that some outcomes have minor clinical interests or effects.**

We conducted sensitivity analysis to evaluate the stability of the results of our meta-analysis, and the results suggested that excluding the small sample studies above had no significant effect on the results of our meta-analysis.

1. Total efficacy. After removing Zhao ^[14]^ , the results still showed that there did have statistical significance in total efficacy between treatment and control group〔OR=4.48，95%CI（3.20，6.26），Z=8.75，P < 0.00001〕

2. Weekly defecation times. After removing Zhao ^[14]^ , the results still showed that there was statistical significance in weekly defecation times changes between baseline and end of treatment between treatment and control group〔MD = 1.01，95%CI（0.75，1.26），Z=7.68，P < 0.00001〕.

3. Defecation time, After removing Zhao ^[14]^ , the results still showed that there was statistical significance in defecation time changes between baseline and end of treatment between treatment and control group〔MD = -3.23，95%CI（-4.24，-2.23），Z=6.32，P < 0.00001〕.

4. Defecation difficulty score. After removing Ma ^[19]^ , The results still showed that was statistical significance in defecation difficulty score changes between baseline and end of treatment between treatment and control group〔MD = -2.18，95%CI（-2.56，-1.81），Z=11.5，P < 0.00001〕.

5. Adverse events of treatment After removing Fu ^[32]^ and Li^[35]^, The results still showed that there was no statistical significance in the incidence of adverse events between treatment and control group〔OR=0.13，95%CI（0.01，1.19），Z=1.80，P =0.07〕

**4.3	As reviewer understand in Figure 9 X-axis should be OR [LOG] and Y-axis should be standard error. If reviewer misunderstand, please clarify the results.**

We used the RevMan software to make the Funnel plot, which used the effect (OR, RR, RD, SMD, etc.) as the X-axis, the reciprocal of the standard error of the effect 1/SE (logOR) as the Y-axis, the X-axis scale with the true number, and the Y-axis scale with SE (logOR). The dots in the chart represented the studies included. The results were more reliable when the sample size was larger and the standard error was smaller, and the dots were concentrated in the narrow area at the top of the funnel plot. The smaller the sample size, the greater the variance, the greater the fluctuation, and the greater the standard error, then the corresponding dots of these studies will be scattered in the wider area of the lower funnel, thus eventually showing an inverted funnel shape. Otherwise the opposite.

5.	Discussion and conclusion

**5.1	Main point of each outcome indicator after analyzing should be discussed.**

14 studies suggested that the effect of routine treatment combined with different ways of external therapies of traditional Chinese medicine is higher than that of routine treatment, which can improve the efficacy of treatment. Compared with routine treatment group, 7 studies suggested that routine treatment combined with different ways of external therapies of traditional Chinese medicine could increase the weekly defecation times, 6 studies suggested that routine treatment combined with different ways of external therapies of traditional Chinese medicine could shorten defecation time, and 3 studies suggested that routine treatment combined with different ways of external therapies of traditional Chinese medicine could improve the defecation difficulty score and 5 studies reported the life quality of CRF patients with constipation which suggested that routine treatment combined with different ways of external therapies of traditional Chinese medicine could reduce the PAC-QOL score and improve the life quality of patients with chronic renal failure and constipation. At last, 3 the study reported the adverse events occurred in the course of treatment, including intestinal obstruction, hematochezia and allergic reactions characterized by skin redness and itching after auricular point sticking, studies suggested that there was no statistical significance in the incidence of adverse events between two groups.

**5.2	Conclusion demonstrates overclaim results.**

In conclusion, The combination of external therapies of traditional Chinese medicine and routine treatment could achieve an excellent curative effect on constipation in people with CRF. And there were no obvious adverse events in the course of treatment.
